# Characteristics predicting recommendation for familial breast cancer referral in a cohort of women from primary care

**DOI:** 10.1007/s12687-020-00452-w

**Published:** 2020-01-22

**Authors:** Siang Ing Lee, Nadeem Qureshi, Brittany Dutton, Joe Kai, Stephen Weng

**Affiliations:** Division of Primary Care, School of Medicine, University of Nottingham, Tower Building, University Park, Nottingham, NG7 2RD UK

**Keywords:** Genetic predisposition to disease, Breast neoplasm, Hereditary cancer, Primary health care

## Abstract

Family history of breast and related cancers can indicate increased breast cancer (BC) risk. In national familial breast cancer (FBC) guidelines, the risk is stratified to guide referral decisions. We aimed to identify characteristics associated with the recommendation for referral in a large cohort of women undergoing FBC risk assessment in a recent primary care study. Demographic, family history, psychological and behavioural factors were collected with family history questionnaires, psychological questionnaires and manual data extraction from general practice electronic health records. Participants were women aged 30–60 with no previous history of breast or ovarian cancer. Data from 1127 women were analysed with stepwise logistic regression. Two multivariable logistic models were developed to predict recommendations for referral: using the entire cohort (*n* = 1127) and in a subgroup with uncertain risks (*n* = 168). Model performance was assessed by the area under the receiver operating curve (AUC). In all 1127 women, a multivariable model incorporating five family history components (BC aged < 40, bilateral BC, prostate cancer, first degree relative with ovarian cancer, paternal family history of BC) and having a mammogram in the last 3 years, performed well (AUC = 0.86). For the 168 uncertain risk women, only paternal family history of BC remained significant (AUC = 0.71). Clinicians should pay particular attention to these five family history components when assessing FBC risk, especially prostate cancer which is not in the current national guidelines.

**Electronic supplementary material:**

The online version of this article (10.1007/s12687-020-00452-w) contains supplementary material, which is available to authorized users.

## Introduction

Breast cancer is the most common cancer in women (World Cancer Research Fund 2018). In developed countries, one in eight women will develop breast cancer in their lifetime (American Cancer Society 2019; Cancer Research UK 2018a, b). Family histories of breast and related cancers are recognised risk factors and have been used to stratify disease risk. Around 5–10% of breast cancers are caused by an inherited faulty genes such as the *BRCA1* and *BRCA2* genes (Cancer Research UK 2014; Cancer Research UK 2018a, b).

For patients at familial risk of breast cancer preventive measures such as risk-reducing prophylactic surgery, chemoprevention or increased surveillance can reduce cancer incidence and/or mortality (Carbine et al. 2018; Cuzick et al. 2013; Domchek et al. 2010; Duffy et al. 2013). In England, the National Institute for Health and Care Excellence (NICE) guideline for identification and management of familial breast cancer risk includes referral criteria for general practitioners (NICE 2013). Familial breast cancer risk assessment enables primary care clinicians to identify patients who meet the criteria for specialist referral to access further assessment and management, which may include genetic counselling, genetic testing and preventive measures.

The NICE guideline, as well as classifying women into high and moderate risk with clear management plans, classifies a significant minority of women with *uncertain risk.* Rather than a direct referral, NICE recommends seeking advice from secondary care specialists on the management/referral of these women (NICE 2013). The challenge of familial breast cancer risk assessment in primary care is further compounded by the lack of detailed cancer family history recording in primary care records (Murff et al. 2004; Tyler and Snyder 2006).

In a recent study that evaluated a systematic approach to identify women at risk of familial breast cancer in primary care, using postal family history questionnaires sent to women aged 30 to 60, one in seven women was assessed as having *uncertain risk* (NIHR SPCR 2013; Chorley et al. 2016). Obtaining specialist opinion for these patients would be a significant workload for both general practitioners and specialists. It also delays patients from receiving appropriate reassurance or specialist care which may cause anxiety and result in presentation in primary care and subsequent possible inappropriate referral to secondary care.

In this study, we explored the demographic, family history, psychological and behavioural factors that are associated with the recommendation for specialist referral for familial breast cancer, with the aim to improve primary care decision-making when managing women with family history of breast and related cancers.

## Materials and methods

This is an observational cohort study with demographic, family history, psychological and behavioural factors collected from patients using a validated family history questionnaire, psychological questionnaires and manual data extraction from general practice electronic health records (NIHR SPCR 2013; Qureshi et al. 2005). We used the patient cohort from a recent study on familial breast cancer risk assessment in primary care: eight general practices in East Midlands were recruited; recruits were women aged 30–60 with no previous personal history of breast or ovarian cancer (NIHR SPCR 2013). Familial breast cancer risk was assessed based on family history, against the NICE guideline recommendations, using a validated family history questionnaire, the FAHRAS decision support software and specialist advice (for those with uncertain risk) (Gorman et al. 2014). For this secondary analysis, we used the data for 1127 participants recruited systematically and undergoing full risk assessment between dates June 2014 and January 2015.

The dependent variable was the final recommended clinical pathway for the participants following the risk assessment: *refer to secondary care* (event) or *manage in primary care* (no event). Box 1 lists the independent variables. The family history data were recoded into variables according to the NICE familial breast cancer referral criteria, such as bilateral breast cancer, male breast cancer, paternal family history of breast cancer, number of first- or second-degree relatives affected and age of diagnosis (NICE 2013). Participant’s occupation was recoded using the UK Office for National Statistics occupation classification using the Cascot software, a computer-assisted structured coding tool (Office for National Statistics 2010; Warwick Institute for Employment Research 2018).

**Box 1:** Independent variables

Demographics • Age (years) • Index of Multiple Deprivation (quintiles) • Ethnicity • Education • Occupation • General practice Family history • Total number of FDR (continuous) • Total number of SDR (continuous) • Number of FDR with breast or ovarian cancer (0 vs = > 1, categorical) • Number of FDR with breast cancer (0 vs = > 1, categorical) • Number of FDR with ovarian cancer (0 vs 1, categorical) • Number of SDR with breast or ovarian cancer (0 vs = > 1, categorical) • Number of paternal relatives with breast cancer (0 vs = > 1, categorical) • Number of paternal relatives with ovarian cancer (0 vs = > 1, categorical) • Number of paternal relatives with breast and ovarian cancer (0 vs 1, categorical) • Number of relatives with breast or ovarian cancer (0 vs = > 1, categorical) • Number of relatives with breast and ovarian cancer (0 vs 1, categorical) • Number of relatives with bilateral breast cancer (0 vs = > 1, categorical) • Number of male relatives with breast cancer (0 vs 1, categorical) • Number of relatives with breast cancer diagnosed under age 40 (0 vs = > 1, categorical) • Number of FDR with breast cancer diagnosed under age 40 (0 vs 1, categorical) • Number of SDR with breast cancer diagnosed under age 40 (0 vs = > 1, categorical) • Number of relatives with prostate cancer (0 vs = > 1, categorical) • Number of paternal relatives with prostate cancer (0 vs = > 1, categorical) • Number of maternal relatives with prostate cancer (0 vs = > 1, categorical) • Number of FDR with prostate cancer (0 vs = > 1, categorical) • Number of relatives with colorectal cancer (0 vs = > 1, categorical) • Number of paternal relatives with colorectal cancer (0 vs = > 1, categorical) • Number of maternal relatives with colorectal cancer (0 vs = > 1, categorical) • Number of FDR with colorectal cancer (0 vs 1, categorical) • Number of relatives with any cancer (continuous) •Number of relatives with sarcoma/adrenal/brain cancer (0 vs = > 1, categorical) • Number of relatives with non-breast cancer diagnosed under age 40 (0 vs 1, categorical) • Number of relatives that died of cancer (0 vs = > 1, categorical) • Number of relatives that died of cancer aged < 40 (0 vs 1, categorical) • Ratio of FDR with breast cancer to total FDR (continuous) • Ratio of FDR with breast/ovarian cancer to total FDR (continuous) • Ratio of SDR with breast/ovarian cancer to total SDR (continuous) Psychological factors (baseline patient questionnaire) • Lerman Breast Cancer Worry (categorical), Cancer Worry Impact Index (continuous) • State-Trait Anxiety Index (continuous) • Positive and Negative Affect Schedule (continuous) • Short Form–Six Dimensions (categorical) • General health (categorical) • Perceived risk of breast cancer (categorical) • Medical Interview Satisfaction Scale (continuous) Health behaviours • Breast self-examination advice from the GP (GP records, yes/no) • Breast self-examination, frequency (patient self-reported) • Breast related GP consultation (GP records, yes/no) • National Health Service health check last 5 years (GP records, yes/no) • Combined oral contraception prescribed (GP records, yes/no) • Progesterone only oral contraception prescribed (GP records, yes/no) • Hormonal replacement therapy prescribed (GP records, yes/no) • Ever had mammogram (patient self-reported, yes/no) • Last mammogram (≤ 3 years, > 3 years, patient self-reported) • Mammogram/ultrasound breast last 12 months (GP records, yes/no)	

*FDR* first-degree relative, *GP* general practitioner, *SDR* second-degree relative, *vs* versus

For continuous data, we conducted median imputation for missing data; for categorical data, missing data were recoded as a separate category of ‘not reported’.

### Statistical analysis

STATA version 13.1 was used for the statistical analysis. *p* < 0.05 was considered significant. Bonferroni correction was used for psychological measures with multiple items to account for correlation between measures.

Demographics for non-participating women were obtained by electronic search from anonymised primary care health records. Age (median) and ethnicity (white and non-white) were compared between participating and non-participating women using two-sample Wilcoxon rank-sum test and Pearson Chi-square test respectively.

Univariate analysis was performed using logistic regression for continuous and binary categorical variables. For non-binary categorical variables, likelihood ratio test was performed against a base model that included age and Index of Multiple Deprivation (IMD) as a priori demographic variables.

Significant variables were then included in the multivariable analysis, with age and IMD quintiles considered a priori. Variables were then removed in stepwise backward elimination to give the final model predicting a recommendation of referral to secondary care. The final model was then applied to the subgroup of participants who were in the uncertain risk group. The area under the receiver operating curve (AUC) was generated to test model performance, a measure of discriminatory accuracy of the model to distinguish between outcome events.

## Results

### Demographics of the study population

From the initial electronic records search, 10,080 women were potentially eligible based on the inclusion criteria. After further review of eligibility by general practitioners, 7012 women were invited to participate; 1127 (16%) consented and completed the familial breast cancer risk assessment in primary care. Reasons for exclusion by general practitioners were unknown due to data protection and privacy laws. Ethnicity and age data for the 8953 potentially eligible women who did not join the study were extracted from the primary care records. These non-participants included both those that were not invited to participate (3068) and those who declined invitation to participate (5885). It was not possible to distinguish from the anonymised non-participant data those who were not invited.

The median time from participants returning the family history questionnaire (risk assessment) to participants receiving an outcome letter (refer or manage in primary care) was 27 days (interquartile range 15 to 43 days). The demographics for the 1127 participants are presented in Table 1. There was a relatively equal spread across all types of education and occupation. Most participants were in the less deprived IMD quintiles (50% in the 4th and 5th quintiles). As the number of non-white participants was very small (2%), they were combined into a single category. There was one of European Jewish ancestry, and three of mixed ethnicity with Jewish ancestry; only one was recommended referral.

**Table 1:** Demographics of the 1127 participants

	*n* (%)
Participants	1127
Age	
Median (IQR)	46 (40–53)
Ethnicity	
White	1099 (98)
Non-white	22 (2)
Not reported	6 (1)
General practice	
A	452 (40)
B	203 (18)
C	43 (4)
D	429 (38)
Education	
School level	169 (15)
Vocational education	106 (9)
College level	210 (19)
Higher education	269 (24)
Other	264 (23)
No formal qualification/still studying	101 (9)
Not reported	8 (1)
Occupation, ONS classification	
Managers, directors, senior official	71 (6)
Professional occupations	218 (19)
Associate professional and technical occupations	127 (11)
Administrative and secretarial occupations	171 (15)
Skilled trades occupations	19 (2)
Personal service occupations	113 (10)
Sales and customer service occupations	50 (4)
Process, plant and machine operatives	9 (1)
Elementary occupations	52 (5)
Student/housewife/unemployed	152 (13)
Not reported	145 (13)
IMD (quintiles)	
Most deprived 1	114 (10)
2	144 (13)
3	253 (22)
4	277 (25)
Least deprived 5	277 (25)
Not reported	62 (6)

*IMD* Index of Multiple Deprivation, *IQR* interquartile range, *ONS* Office for National Statistics

*NB* non-white ethnicity included Black, Indian subcontinent, Mediterranean, European Jewish, Brazilian, Philippine and mixed ethnicities

For the 8953 non-participating women, the median age was 46 years (interquartile range 39–52); 37% (3271/8953) had no ethnicity recorded in their primary care records. Despite this being a known problem with routinely collected primary care data sources, primary care ethnicity data are generally comparable with the UK census (Mathur et al. 2014). White ethnicity comprised 97% (5509/5682) of non-participating women with recorded ethnicity, similar to the ethnicity profile for the borough of the participating general practices (96%) (Office for National Statistics 2012). Comparing the 1127 participants with the non-participants, although there was a significant difference for age (*p* = 0.003), both groups had a median age of 46 years. For those with reported ethnicity, participation rate was 17% (1099/6608) for white participants and 11% (22/195) for non-white participants; this difference was of borderline significance (*p* = 0.047).

### Risk assessment outcomes: refer or manage in primary care

**Fig. 1:**
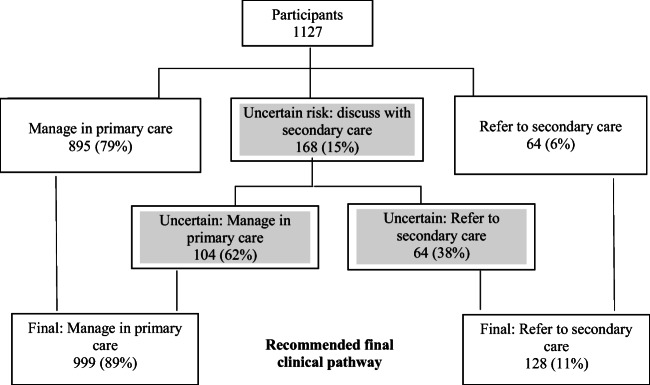
Recommended clinical pathway for 1127 participants after familial breast cancer risk assessment and discussion with secondary care, uncertain risk group highlighted in grey

Of the 1127 participants, 168 needed discussion with secondary care (uncertain risk), of which 64 (38%) were recommended referral (Fig. 1). Twenty-eight of the uncertain risk cases were identified by the research team beyond that of the decision support software, of which 12 were recommended referral after discussion with secondary care. The final pathway for all participants was the following: refer to secondary care for 128 participants and manage in primary care for 999 participants.

### Univariate analysis: factors associated with recommendation for referral

From the univariate analysis of all factors listed in Box 1, six variables were significant and included in the multivariable model: five family history variables (breast cancer diagnosed aged < 40, bilateral breast cancer, prostate cancer, first-degree relatives (FDR) with ovarian cancer, paternal family history of breast cancer) and one health behaviour variable (last mammogram, Supplementary material 1, 2). The variable ‘breast cancer aged < 40’ had the strongest association with recommendation for referral (OR 26.44, 95% CI 12.44 to 56.19). A small number of participants (*n* = 16) with family history of non-FDR breast cancer aged < 40 did not meet the NICE referral criteria; they were given the ‘Uncertain’ category and after discussion with the specialists, referral was not indicated for 10 participants.

### Multivariable regression models

In the multivariable analysis including all 1127 participants, the family history variable with the strongest association of recommendation for referral was breast cancer in younger women (aged < 40, AOR 22.12, 95% CI 9.36 to 52.30), followed by bilateral breast cancer, relevant cancers (prostate cancer, FDR with ovarian cancer) and breast cancer in the paternal lineage (Table 2).

**Table 2:** Multivariable model predicting recommendation of secondary care referral for 1127 participants undergoing familial breast cancer risk assessment in primary care, *p* < 0.0001

Variables	Adjusted OR	95% CI	*p* value
Age	0.97	0.93 to 1.01	0.11
IMD (quintiles)			
Most deprived, 1	Reference		
2	0.56	0.22 to 1.40	0.21
3	0.67	0.31 to 1.47	0.32
4	0.59	0.27 to 1.31	0.19
Least deprived, 5	0.84	0.39 to 1.82	0.66
Not available	0.82	0.29 to 2.31	0.71
Last mammogram			
≤ 3 years	Reference		
> 3 years	0.27	0.08 to 0.91	0.04
Not reported	0.32	0.16 to 0.63	0.001
Family history of breast cancer aged < 40	22.12	9.36 to 52.30	< 0.001
Family history of bilateral breast cancer	12.36	5.31 to 28.80	< 0.001
Family history of prostate cancer	7.45	3.98 to 13.95	< 0.001
FDR with ovarian cancer	5.56	2.43 to 12.69	< 0.001
Paternal family history of breast cancer	3.79	2.22 to 6.47	< 0.001

*CI* confidence interval, *FDR* first-degree relative, *IMD* Index of Multiple Deprivation, *OR* odds ratio

Participants whose last mammogram was more than 3 years ago or who did not report their last mammogram were less likely to be recommended a secondary care referral (AOR 0.27, 95% CI 0.08 to 0.91; AOR 0.32, 95% CI 0.16 to 0.63 respectively). Of these 680 participants who did not have/report a mammogram in the last 3 years, only 9% were not compliant, the rest were not in the national breast cancer screening age group of 50–70.

Combining these significant factors with age and IMD quintiles a priori produced a predictive model with high discriminatory accuracy (AUC 0.86, Supplementary material 3). When this multivariable model was applied to the subgroup of 168 women with uncertain risk, the discrimination was reduced by 15% (AUC 0.71, Supplementary material 4). Further, the only variable that remained significant was family history of breast cancer in the paternal lineage (Table 3).

**Table 3:** Multivariable model predicting recommendation of secondary care referral for 168 uncertain risk participants undergoing familial breast cancer risk assessment in primary care, *p* = 0.01

Variables	Adjusted OR	95% CI	*p* value
Age	0.96	0.90 to 1.02	0.16
IMD (quintiles)			
Most deprived, 1	Reference		
2	0.64	0.16 to 2.53	0.53
3	1.15	0.37 to 3.57	0.81
4	0.76	0.23 to 2.58	0.67
Least deprived, 5	1.98	0.63 to 6.24	0.24
Not available	0.47	0.08 to 2.80	0.41
Last mammogram			
≤ 3 years	Reference		
> 3 years	0.34	0.05 to 2.18	0.26
Not reported	0.47	0.16 to 1.37	0.17
Paternal family history of breast cancer	5.08	2.06 to 12.54	< 0.001
Family history of breast cancer aged < 40	0.61	0.17 to 2.17	0.45
Family history of bilateral breast cancer	0.58	0.18 to 1.85	0.35
Family history of prostate cancer	1.84	0.75 to 4.51	0.18
FDR with ovarian cancer	0.79	0.30 to 2.05	0.63

*CI* confidence interval, *FDR* first-degree relative, *IMD* Index of Multiple Deprivation, *OR* odds ratio

## Discussion

### Main findings and comparison with existing literature

This study confirmed the importance of certain family history components when assessing familial breast cancer risk in primary care, namely breast cancer diagnosed age < 40, in both breasts, or in the paternal lineage and related cancers such as ovarian cancer in FDR and prostate cancer. These family history components held the greatest weight in deciding whether referral was recommended by a decision support software based on clinical guidelines. There was less clarity on the predictors of referral for women with uncertain risk. As a decision support software based on clinical guidelines was used to decide whether referral was indicated or not, it was not surprising that psychological factors did not influence referral recommendations.

These family history components are known risk factors for breast cancer. A Lancet study which combined 52 epidemiological studies found that the excess lifetime incidence of breast cancer for women with one relative affected before age 40 compared with over age 60 is 3.8% (Collaborative Group on Hormonal Factors in Breast Cancer 2001). Bilateral breast cancer and ovarian and prostate cancers are thought to be associated with pathogenic variants in the *BRCA1* and *BRCA2* genes (Petrucelli et al. 1998).

Family history of prostate cancer, which was identified as a significant factor, is currently not included in clinical guidelines (NICE 2013). This is significant as a pathogenic variant in the *BRCA2* mutation gene in particular which is linked to familial breast cancer also increases the risk of prostate cancer (Cancer Research UK 2018a, b). The Women’s Health Initiative observational study of over 70,000 women showed that prostate cancer in a FDR increased a woman’s risk of developing breast cancer (adjusted hazard ratio 1.14); this increases to 78% when there is a family history of breast and prostate cancers (Beebe-Dimmer et al. 2015).

### Strengths and limitations

To our knowledge, this is the first study to evaluate factors associated with referral recommendations for women undergoing familial breast cancer risk assessment in primary care, as defined by clinical guidelines. The strength of this study lies in the large sample size of 1127 participants who engaged with the full familial breast cancer assessment. However, the uptake rate in the original study was low, leading to responder bias where women with a family history of breast cancer may be more motivated to participate. Nevertheless, this model would be applicable to a real-world setting where uptake may be low when women were systematically invited for a familial breast cancer risk assessment.

Comparisons of demographics were made between participants and non-participants. The research team received anonymised data from all potentially eligible women, but it was not possible to identify from these women who were excluded from being eligible in study participation by the general practitioners due to privacy and data protection laws.

The availability of multiple data sources on the same person enabled the exploration of a range of family history, psychological, behavioural, sociodemographic and clinical factors. In-depth analysis of the family history was made possible by the quality of the data collected using a structured, validated family history tool (Qureshi et al. 2005). The psychological measures were also assessed using validated tools such as the Lerman Breast Cancer Worry and State-Trait Anxiety Index (Lerman et al. 1991; Spielberger et al. 1983).

We were not able to account for socioeconomic variation at the general practice level as the small number of practices (*n* = 4) did not allow for a cluster effect analysis. Nevertheless, socioeconomic variation at participant level was measured using the IMD. The study population was predominantly white and above average on the socioeconomic gradient. The mammography uptake in this cohort suggests a responders bias, where women who are more concerned of their familial breast cancer risk are more likely to participate in the study. This, together with the lack of ethnic and socioeconomic variation, limits the generalisation of findings to other population groups.

To strengthen the multivariable model, we used the 1127 cohort before applying it to the smaller uncertain risk group, as the latter was too small to develop a multivariable model. As a general rule of thumb, logistic regression for prognostic modelling requires approximately 10 outcome events per predictor (Peduzzi et al. 1996). Similarly, the response rates in certain categories were too small and had to be combined or excluded from the analysis. For instance, Jewish ancestry, a known risk factor for familial breast cancer, was not used as a covariate because there were only four participants.

### Future research recommendations

Several breast cancer risk prediction models such as the Gail model and the Tyrer-Cuzick model included other breast cancer risk factors, for instance, weight and age of menarche (Amir et al. 2003). These were not available for our analysis as the original study focused on familial risk. These risk factors are likely to be important predictors of the need for specialist assessment for breast cancer risk and should be considered in future research. To illustrate this, the PROCAS study compared the number of breast cancers detected using NICE guidelines versus the Tyrer-Cuzick model for familial breast cancer risk assessment in women attending routine mammographic screening; the Tyrer-Cuzick model classified more women at risk (8.8% versus 3.7%) and detected more with breast cancer (8 versus 5 of 37 cases detected) (Evans et al. 2014).

The exploratory nature of this study may result in type 1 error: confirmation of the finding in a different dataset would be desirable. The multivariable model also needs to be tested on longer term outcomes, such as the outcomes after secondary care assessment or cancer incidence. The uncertain risk group needs more investigation through other cohort studies or qualitative methods involving clinicians to explore what determines referral recommendations.

## Conclusion and clinical implications

Clinical guideline recommends taking a comprehensive family history when assessing familial breast cancer risk (NICE 2013). This can be challenging for primary care clinicians who have limited consultation time. This study confirmed the salient family history components that primary care clinicians need to pay particular attention to when identifying patients who need secondary care referral. Prostate cancer family history needs to be considered when assessing a woman’s familial breast cancer risk, in addition to the well-known risk factors of breast and ovarian cancer family history.

## Electronic supplementary material

ESM 1 (DOCX 37 kb).
