# Supplementary material for: Characteristics predicting recommendation for familial breast cancer referral in a cohort of women from primary care
Source: J Community Genet. 2020 Jan 22;11(3):331–8. doi: 10.1007/s12687-020-00452-w (PMC7295867; doi:10.1007/s12687-020-00452-w)
Supplement: Supplementary file 1 — (DOCX 37 kb). [file 12687_2020_452_MOESM1_ESM.docx]

**Supplementary material 1**

The number of participants with the significant univariates by recommendation to refer / manage in primary care, for all 1127 participants and 168 *uncertain risk* participants

| **All participants** | **Refer, n (%)** | **Manage in primary care, n (%)** | **Total, n (%)** |
| --- | --- | --- | --- |
| Total number | 128 | 999 | 1127 |
| IMD (quintiles) |  |  |  |
| Most deprived, 1 | 18 (14) | 96 (10) | 114 (10) |
| 2 | 13 (10) | 131 (13) | 144 (13) |
| 3 | 29 (23) | 224 (22) | 253 (22) |
| 4 | 28 (22) | 249 (25) | 277 (25) |
| Least deprived, 5 | 30 (23) | 247 (25) | 277 (25) |
| Not available | 10 (8) | 52 (5) | 62 (6) |
|  |  |  |  |
| Last mammogram |  |  |  |
| <=3 years | 65 (51) | 382 (38) | 447 (40) |
| > 3 years | 7 (5) | 68 (7) | 75 (7) |
| Not reported | 56 (44) | 549 (55) | 605 (54) |
|  |  |  |  |
| Family history of breast cancer aged <40 | 27 (21) | 10 (1) | 37 (3) |
| Family history of bilateral breast cancer | 22 (17) | 13 (1) | 35 (3) |
| Family history of prostate cancer | 30 (23) | 37 (4) | 67 (6) |
| FDR with ovarian cancer | 15 (12) | 22 (2) | 37 (3) |
| Paternal family history of breast cancer | 42 (33) | 81 (8) | 123 (11) |
| **Uncertain risk participants** | **Refer, n (%)** | **Manage in primary care, n (%)** | **Total, n (%)** |
| Total number | 64 | 104 | 168 |
| IMD (quintiles) |  |  |  |
| Most deprived, 1 | 8 (13) | 15 (14) | 23 (14) |
| 2 | 6 (9) | 17 (16) | 23 (14) |
| 3 | 15 (23) | 27 (26) | 42 (25) |
| 4 | 10 (16) | 21 (20) | 31 (18) |
| Least deprived, 5 | 22 (34) | 17 (16) | 39 (23) |
| Not available | 3 (5) | 7 (7) | 10 (6) |
|  |  |  |  |
| Last mammogram |  |  |  |
| <=3 years | 28 (44) | 42 (40) | 70 (42) |
| > 3 years | 3 (5) | 7 (7) | 10 (6) |
| Not reported | 33 (52) | 55 (53) | 88 (52) |
|  |  |  |  |
| Family history of breast cancer aged <40 | 6 (9) | 10 (10) | 16 (10) |
| Family history of bilateral breast cancer | 7 (11) | 13 (13) | 20 (12) |
| Family history of prostate cancer | 15 (23) | 16 (15) | 31 (18) |
| FDR with ovarian cancer | 8 (13) | 22 (21) | 30 (18) |
|  |  |  |  |
| Paternal family history of breast cancer | 26 (41) | 16 (15) | 42 (25) |

FDR: first degree relative, IMD: Index of Multiple Deprivation

**Supplementary material 2**

Factors found to be significant in the univariate analysis for 1127 participants with age and IMD quintiles as *a priori*

|  | **Univariate** | | |
| --- | --- | --- | --- |
| **Variable** | **OR** | **95% CI** | **P value** |
| Age | 1.00 | 0.98 to 1.03 | 0.80 |
| IMD (quintiles) |  |  |  |
| Most deprived, 1 | reference | |  |
| 2 | 0.53 | 0.25 to 1.13 | 0.10 |
| 3 | 0.69 | 0.37 to 1.30 | 0.25 |
| 4 | 0.60 | 0.32 to 1.13 | 0.12 |
| Least deprived, 5 | 0.65 | 0.34 to 1.22 | 0.18 |
| Not available | 1.03 | 0.44 to 2.38 | 0.95 |
|  |  |  |  |
| Last mammogram |  |  |  |
| <=3 years | reference | |  |
| > 3 years | 0.60 | 0.27 to 1.38 | 0.23 |
| Not reported | 0.60 | 0.41 to 0.88 | 0.01 |
|  |  |  |  |
| Family history of breast cancer aged <40 | 26.44 | 12.44 to 56.19 | <0.0001 |
| Family history of bilateral breast cancer | 15.74 | 7.71 to 32.16 | <0.0001 |
| Family history of prostate cancer | 7.96 | 4.71 to 13.45 | <0.0001 |
| FDR with ovarian cancer | 5.90 | 2.97 to 11.69 | <0.0001 |
| Paternal family history of breast cancer | 5.53 | 3.59 to 8.54 | <0.0001 |

CI: confidence interval, FDR: first degree relative, IMD: Index of Multiple Deprivation, OR: odds ratio

**Supplementary material 3**

Receiver operating curve (ROC) for multivariable model predicting recommendation of secondary care referral for 1127 participants undergoing familial breast cancer risk assessment in primary care

**Supplementary material 4**

Receiver operating curve (ROC) for multivariable model predicting recommendation of secondary care referral for 168 uncertain risk participants undergoing familial breast cancer risk assessment in primary care ****
